# Management of Hypothyroidism in Internal Medicine: Patient Profile and Effects of an Educational Programme in the Cluster-Randomized FADOI TIAMO Study

**DOI:** 10.3389/fendo.2022.839300

**Published:** 2022-06-13

**Authors:** D. Brancato, B. Biondi, T. M. Attardo, A. Fierro, M. Nizzoli, R. Vettor, E. Monaco, R. Romano, V. Ariete, C. Usai, E. Zagarrì, M. Campanini

**Affiliations:** ^1^ Internal Medicine Department, Ospedale Civile di Partinico, Palermo, Italy; ^2^Department of Clinical Medicine and Surgery, School of Medicine and Surgery, University of Naples Federico II, Naples, Italy; ^3^Internal Medicine Department, Ospedale di Circolo e Fondazione Macchi, Varese, Italy; ^4^Formerly at Internal Medicine Department, Pertini Hospital, Rome, Italy; ^5^Department of Endocrinology and Metabolic Diseases, Morgagni - Pierantoni Hospital, Forlì, Italy; ^6^Department of Medicine, Università degli Studi, Padova, Italy; ^7^Internal Medicine Department, San Paolo Hospital, Savona, Italy; ^8^Geriatric Department, Azienda Ospedaliera di Rilievo Nazionale e Alta Specializzazione “Garibaldi”, Catania, Italy; ^9^Internal Medicine Department, Gubbio - Gualdo Tadino Hospital, Perugia, Italy; ^10^Internal Medicine Department, S.S. Annunziata Hospital, Sassari, Italy; ^11^Department of Clinical Research, FADOI Study Center, Milano, Italy; ^12^Internal Medicine Department, Maggiore della Carità Hospital, Novara, Italy

**Keywords:** hypothyroidism, levo-thyroxine therapy, internal medicine, guidelines, educational program

## Abstract

**Background and aims:**

There is still limited knowledge regarding the clinical profile and appropriateness of treatment in patients with hypothyroidism hospitalized in Internal Medicine (IM) Departments in Italy. The aim of this study is to evaluate: 1) the characteristics of patients and possible deviations from national and international clinical practice recommendations (CPRs) in evidence-based guidelines (EBGs); 2) the improvement of patient management by means of a standardized educational programme (EP).

**Methods:**

A nationwide multicentre study, comprising two replications of a retrospective survey (phases 1 and 3) with an intervening EP (phase 2) in half of the centres and no EP in the other half, was conducted. The EP was based on outreach visits. Centres were assigned to the two arms of the study, labelled the training group (TG) and control group (CG) respectively, by cluster randomization. Four EBGs and 39 CPRs provided the basis on which 22 treatment management indicators were identified (7 referring to the time of hospital admission, 15 to post-admission).

**Results:**

The 21 participating centres recruited 587 hospitalized patients with hypothyroidism, 421 of which were females (71.7%, mean age 74.1 + 14.4 yrs): 318 in phase 1 and 269 in phase 3. The cause of hypothyroidism was unknown in 282 patients (48%). Evaluation at the time of admission identified satisfactory adherence to CPRs (>50%) for 63.6% of the indicators. In the phase 3, TG centres showed significant improvement vs CG in 4 of the 15 post-admission indicators, while 1 out of 15 was significantly worse.

**Conclusions:**

The EP based on outreach visits significantly improved some indicators in the management of patients with hypothyroidism, with specific reference to appropriateness of TSH dosage and levothyroxine (LT4) treatment modality.

**Clinical Trial Registration:**

ClinicalTrials.gov, identifier NCT05314790.

## Introduction

Prevalence of overt hypothyroidism in the general population varies according to iodine intake, from 0.3% to 3.7% in the USA and 0.2% to 5.3% in Europe (1, 2). However, it is estimated that subclinical hypothyroidism, as defined by elevated TSH with FT4 within the normal range, has a prevalence of up to 10% in the adult population (3). Prevalence of overt and subclinical hypothyroidism increases with age, becoming as high as 20% in elderly subjects (4); it is among the most common conditions in multimorbid patients (5). It is therefore reasonable to posit that hypothyroidism is very common in the typically elderly, and often fragile, inpatients of Internal Medicine (IM) departments, among whom multimorbidity and polytherapy are preponderant (6). It should also be borne in mind that there is no universal consensus on the definition of hypothyroidism in these patients, its biological significance and related treatment indications (7, 8).

Treatment of hypothyroidism is based on replacement therapy with levothyroxine (LT4), to achieve clinical and biochemical euthyroidism (resolving signs and symptoms, with TSH within the normal range) (9–11). However, data in the literature indicate that 35-60% of patients in treatment do not achieve target TSH values and prove to be either under- or overtreated (10–12), clearly with negative effects in terms of morbidity and mortality. In this regard, the risk that overtreatment can lead to exogenous subclinical hyperthyroidism, with potential cardiovascular complications, is one of the most critical issues in treatment of hypothyroidism, particularly in the elderly (3, 13–16).

Clinical management is, indeed, far from easy. There is a lack of knowledge regarding not only the clinical profile of hypothyroid patients treated by IM departments, but also the importance given by IM specialists to hypothyroidism, with particular reference to its occurrence in elderly patients. In addition, the peculiar profile of the IM patient (pharmacological interferences, polytherapy, altered nutritional status, dysphagia, debilitating acute and/or chronic diseases) means that the real-life IM setting is not conducive to ready implementation of clinical guidelines developed to support treatment decisions for hypothyroidism (9, 10, 17).

For this reason, the Italian Federation of Internal Medicine Hospital Clinicans’ Associations (FADOI) designed and carried out the TIAMO study (*Trattamento dell’Ipotiroidismo nell’Ambito della Medicina interna Ospedaliera*: Treatment of hypothyroidism in IM departments), to examine management of hypothyroidism by IM departments. Focusing on both inpatient and outpatient treatment, the study was organized under the following main headings: 1) clinical profile (aetiology and severity of hypothyroidism, method of treatment, therapeutic target, comorbidities, concomitant treatments); 2) any offset between treatment standards for hypothyroidism and their real-life implementation; 3) the effects of an educational programme (EP), in terms of alignment to standards.

Given the differences between the inpatient and outpatient settings in which the TIAMO study was carried out, the results are presented and discussed below only for the inpatient setting (TIAMO-inpatients substudy).

## Materials and Methods

### Study Design

The TIAMO study, the methodology for which had already been successfully applied in other FADOI study settings (18–20), followed the ‘survey before and after educational programme’ model in the broader context of the Deming cycle. A multicentre observational study, TIAMO was designed for involvement of at least 20 IM departments nationwide, with a view to overall enrolment of 1,200 hypothyroid patients.

The study comprised the following three phases:

- phase 1 - retrospective data collection, to include inpatients and outpatients treated for any reason at IM departments, presenting with a history of hypothyroidism and/or TSH above the normal range, with or without FT4 and/or FT3 below the normal range;
- phase 2 - in half of the participating centres (selected by cluster-randomized allocation, with a 1:1 ratio), implementation of an EP based on meetings within the Department, coordinated by an outside tutor (outreach visit model). The EP content was defined beforehand by a Scientific Board, on the basis of evidence-based guidelines (EBGs) and an up-to-date literature review, but also with a specific focus on any departures from standard clinical practice recommendations (CPRs) identified at each IM department in phase 1;
- phase 3 - about 6 months after the EP, once again comprising retrospective data collection in all centres. The data collection methodology being the same as in phase 1, this made it possible to evaluate any effects of the EP. This was done by identifying any departures from standard CPRs, comparing phase 3 results for the IM centres running an EP (training group - TG) with those for the centres where none was provided (control group - CG); the TG’s results in phase 3 were also compared longitudinally with those in phase 1.

For each IM department, in both phase 1 and phase 3, the study protocol specified a review of the clinical records of the last 15 patients consecutively admitted with a diagnosis of hypothyroidism.

### Selection Criteria

Inclusion criteria were as follows:

- known diagnosis of hypothyroidism on the basis of medical history or laboratory results, as indicated in the clinical record of inpatients admitted to the IM department for any condition;
- age ≥ 18 years.

Female patients were excluded if pregnant or seeking to become pregnant.

### Data Collection, Selection of Recommendations and Generation of Indicators

Data collected during phases 1 and 3 by means of an *ad hoc* electronic case report form were intended to provide general information on the participating centre, the clinical characteristics of the patients enrolled, and compliance with the specific EBGs to which the Scientific Board had given the highest AGREE II score (21) at the time of drafting and implementing the study protocol (9, 10, 17, 22–24). The recommendations selected by the Scientific Board focused on topics of particular importance for IM patients (severity of hypothyroidism, comorbidities, polytherapy, therapeutic compliance, adverse events, hospitalization, critical clinical conditions, advanced age), identified as useful with a view to evaluating standards of care as a basis for generating solid and reliable indicators. These recommendations were grouped under 11 main headings, taking into account the following parameters: severity of hypothyroidism; factors determining initial LT4 dosage; timing of LT4 administration in relation to meals; gastrointestinal conditions, or medication, creating possible pharmacological interference with LT4 absorption; use of LT4 formulations other than tablets (tbts); achievement of a specific TSH target value in relation to age and comorbidities.

The indicators generated by the selected recommendations, listed in **Table 1**, refer to data generated:

1) during assessment at the time of admission (entry indicators), and thus not related to management of the patient by the IM specialist;
2) when the patient had been assessed and was being treated (post-admission indicators), thus reflecting the IM specialist’s management of the case.

**Table 1:** Recommendations and indicators. The selected recommendations are numbered as in the respective publications from which they are drawn.

Recommendations					Indicators	
Subject of recommendations	Guidelines from which the recommendations are drawn				At the time of hospital admission	Post-admission
	ETA 2013 (10)	ATA 2014 (17)	AME 2016 (23)	SIE2016 (24)		
**Monotherapy with LT4**		1	2	1	1.1. Patients on LT4 replacement therapy/Patients on replacement therapy	2.1. See 1.1.
**Use of non-tablet formulations**		2b			1.2.1. Patients on LT4 replacement therapy who are not taking sg or s.o., are on absorption-hindering medication and/or do not comply with timing in relation to meals/Patients on LT4 replacement therapy who do not comply with the prescribed timing before meals and/or are taking absorption-hindering medication	2.2.1 See 1.2.1.
			9, 10, 11			2.2.2. Patients on LT4 sg or s.o. replacement therapy, who do not comply with the prescribed timing before breakfast and/or present with absorption-hindering medication or disease/Patients on LT4 replacement therapy who do not respect the prescribed timing before breakfast and/or present with absorption-hindering medication or disease
		2				2.2.3. Patients on LT4 replacement therapy, with no brand, generic or formulation switches/Patients on LT4 replacement therapy
**Achievement of target TSH**		1b, 6a			1.3.1. Patients on replacement therapy with TSH in the ATA target range (*)/Patients on replacement therapy with known TSH	
			1,3		1.3.2. Patients on replacement therapy with TSH in the AME target range (*)/Patients on replacement therapy with known TSH	
**Factors determining LT4 dosage**		4	5		1.4. Patients on replacement therapy with known body weight/Patients on replacement therapy	
**Timing of LT4 dose in relation to meals**		3	9		1.5.1. Patients on replacement therapy, taking LT4 tbts at least 60’ before breakfast or at least 3 h after dinner/Patients on replacement therapy with LT4 tbts	2.5.1. See 1.5.1.
**Medication interfering with LT4 absorption**		3b	9		1.5.2. Patients on replacement therapy, taking medication interfering with absorption at least 4 h after LT4 tbts/Patients on replacement therapy with LT4 tbts, taking medication interfering with absorption	2.5.2. See 1.5.2.
**GI conditions interfering with LT4 absorption**		3c	9			2.5.3. Patients on replacement therapy requiring high doses of LT4 and subjected to diagnostic procedures for diseases interfering with absorption/Patients on replacement therapy requiring high doses of LT4
**Initial dosage of LT4**		4a, 4b, 6	6			2.6. Elderly and/or cardiopathic patients with newly diagnosed hypothyroidism, starting therapy with LT4 < 50 µg/die/Elderly and/or cardiopathic patients with newly diagnosed hypothyroidism, starting therapy with LT4
**Prescribed TSH**		3b, 4b	7, 13			2.7.1. Patients with newly diagnosed hypothyroidism, starting therapy with LT4 and prescribed TSH after 4-6 weeks/Newly diagnosed patients starting therapy
						2.7.2. Patients with a change of LT4 dosage, prescribed TSH after 4-6 weeks/Patients with a change of LT4 dosage
						2.7.3. Patients with a switch of LT4 brand, generic or formulation, prescribed TSH after 4-6 weeks/Patients with a switch of LT4 brand, generic or formulation,
**Specific recommendations for inpatients**		20a, 22b				2.8.1. Inpatients with history of hypothyroidism and recent or post-admission TSH assay/Inpatients with history of hypothyroidism
						2.8.2. Inpatients with NTI syndrome who are not starting therapy with LT4 or LT3/In-patients with NTI syndrome
**Specific recommendations for subclinical hypothyroidism**	2,14,15					2.9.1. Patients with newly diagnosed subclinical hypothyroidism, not immediately prescribed LT4/Patients with newly diagnosed subclinical hypothyroidism
	4,7,8,12,13,15					2.9.2. Patients with newly diagnosed subclinical hypothyroidism, immediately prescribed LT4/Patients with newly diagnosed subclinical hypothyroidism, immediately prescribed LT4 a/c to guidelines

ETA, European Thyroid Association; ATA, American Thyroid Association; AME, Associazione Medici Endocrinologi (Italian Endocrinology Association); SIE, Società Italiana di Endocrinologia (Italian Endocrinology Society); tbts, tablets; o.s., oral solution; sg, softgel capsules; L-T4, levothyroxine; TSH, thyrostimulant hormone. (*) Excluding patients with central hypothyroidism from hypothalamic-pituitary disease.

The Scientific Board adopted the following standard definitions:

- “elderly subjects” > 75 years;
- “ high LT4 dosage” is a supraphysiological dose based on body weight, that is > 1.7 µg/kg/die, or in any case at a level suggesting a condition of malabsorption and pharmacological interference;
- “overt primary hypothyroidism” is defined in the presence of primary hypothyroidism already under treatment or when we found elevated TSH, with below-threshold FT4 (or FT4 and FT3 below threshold in the more severe form) at a recent reading < 1 month prior to hospitalization or at hospital admission and persistently confirmed in the subsequent follow-up;
- “subclinical primary hypothyroidism” is identified in the presence of TSH persistently > 4.5 in adults or > 7 mUI/L in elderly subjects, (defined in this study, as aged over 75 years)” (25);
- “central hypothyroidism”, when known from the patient’s history or in the presence of an inappropriately low TSH value associated with FT4 below the normal range, especially if associated with radiological evidence of hypothalamic-pituitary disease;
- “NTI (non-thyroidal illness) syndrome”, identified on clinical basis, severity of intercurrent illness and with TSH < the upper limit, with FT3 < the lower limit, and no stated history or radiological evidence of central hypothyroidism from hypothalamic-pituitary disease.
- “exogenous subclinical hyperthyroidism” in patients receiving LT4: TSH < 0.4 mUI/L; FT4 and FT3 within the normal range (26).

### Sample Size and Analysis of Results

Given the study’s exploratory nature and the lack of a realistic basis for well-defined initial hypotheses, a formal calculation was not made to identify the sample size required for purposes of comparison (TG vs CG in phase 3; phase 3 vs phase 1 in the TG).

However, the sample size envisaged was considered adequate for the required preliminary observation and subsequent statistical inferencing. The purpose of the preliminary observation was to describe the population and the real-life practices applied to management of hypothyroidism by IM facilities. For data analysis, continuous variables in descriptive statistics were indicated as mean or median values and related distribution parameters, while categorical variables were indicated as a numerical count and/or percentage (n, %). Statistical analysis was based on parametric tests where distribution was normal (Student’s t-test for unpaired data), and non-parametric tests elsewhere (Mann-Whitney U test for unpaired data). For statistical analysis with nominal variables, the chi^2^ test was used, except when at least one of the groups comprised fewer than 6 subjects, in which case Fisher’s exact test was used. Data distribution was assessed by the Kolmogorov-Smirnov test. Statistical significance was set at 5%. All the comparisons were performed using Kyplot 5.0 (KyensLab Inc.).

### Study Promoter and Data Ownership

The study was promoted by the FADOI Foundation’s Clinical Research Department (*Fondazione FADOI - Dipartimento per la Ricerca Clinica “Centro Studi”*), a not-for-profit organization which was also responsible for the related scientific and operational coordination. The FADOI Foundation is also the data owner.

### Ethical Considerations

The study was run in compliance with the Declaration of Helsinki, with current clinical trial regulations and with good clinical practices. The protocol was approved by the Institutional Review Board of the coordinating centre (*Presidio Ospedaliero dell’Ospedale Civico di Partinico*), as well as by the various participating centres’ respective ethics committees.

## Results

Phase 1 data collection ran from June to October 2016, on consecutive patients hospitalized before June 2016. Phase 2 outreach visits ran through April and May 2017. Phase 3 data were collected in December 2017 and January 2018, for consecutive patients hospitalized before December 2017, after completion of outreach visits.

A total of 21 IM departments were involved, with a nationwide distribution: 11 were randomly assigned to the TG, and 10 to the CG.

Data assessment was carried out from 2018-2020.

### General Characteristics of the Study Population

**Figure 1:**
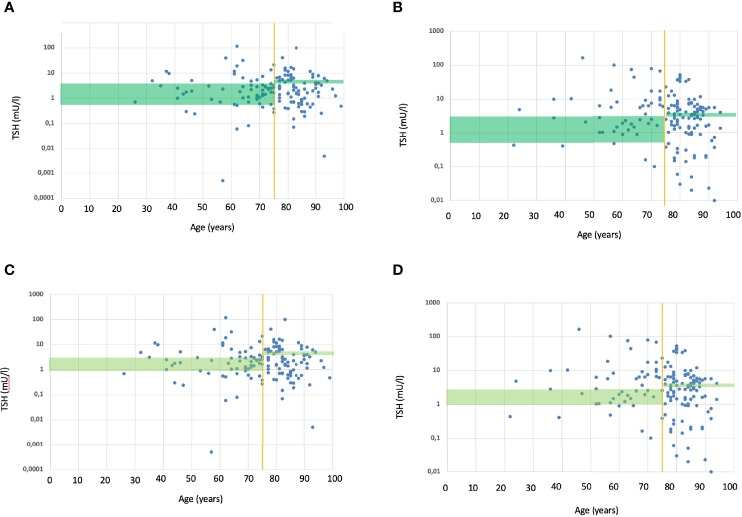
**(A)** TSH values (within and outside ATA target range) in relation to age, in subjects treated with L-T4 during the phase 1. The green rectangle shows the ATA TSH target range (17), respectively for non-elderly (to the left of the yellow vertical line) and elderly subjects (to the right of the yellow vertical line). Each blue dot represents the TSH value of a single subject treated with L-T4. Overall, 23.8% subjects where within ATA TSH target range; only few elderly subjects were within the target. **(B)** TSH values (within and outside ATA target range) in relation to age, in subjects treated with L-T4 during the phase 3. The green rectangle shows the ATA TSH target range (17), respectively for non-elderly (to the left of the yellow vertical line) and elderly subjects (to the right of the yellow vertical line). Each blue dot represents the TSH value of a single subject treated with L-T4. Overall, 26.0% subjects where within ATA TSH target range; only few elderly subjects were within the target. **(C)** TSH values (within and outside AME target range) in relation to age, in subjects treated with L-T4 during the phase 1. The green rectangle shows the AME TSH target range (23), respectively for non-elderly (to the left of the yellow vertical line) and elderly subjects (to the right of the yellow vertical line). Each blue dot represents the TSH value of a single subject treated with L-T4. Overall, 18.2% subjects where within AME TSH target range; only few elderly subjects were within the target. **(D)** TSH values (within and outside AME target range) in relation to age, in subjects treated with L-T4 during the phase 3. The green rectangle shows the AME TSH target range (23), respectively for non-elderly (to the left of the yellow vertical line) and elderly subjects (to the right of the yellow vertical line). Each blue dot represents the TSH value of a single subject treated with L-T4. Overall, 14.7% subjects where within AME TSH target range; only few elderly subjects were within the target.

**Figure 2:**
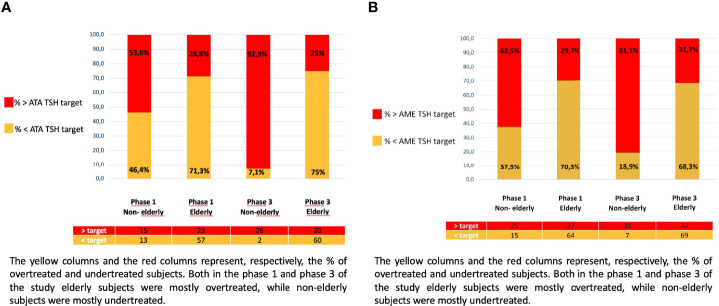
**(A)** Patients under and overtreated in relation to the ATA TSH target, in phase 1 and 3 of the study: elderly and non-elderly. The yellow columns and the red columns represent, respectively, the % of overtreated and undertreated subjects. Both in the phase 1 and phase 3 of the study elderly subjects were mostly overtreated, while non-elderly subjects were mostly undertreated. **(B)** Patients under and overtreated in relation to the AME TSH target, in phase 1 and 3 of the study: elderly and non-elderly. The yellow columns and the red columns represent, respectively, the % of overtreated and undertreated subjects. Both in the phase 1 and phase 3 of the study elderly subjects were mostly overtreated, while non-elderly subjects were mostly undertreated.

The TIAMO-inpatients substudy comprised 587 patients: 318 in phase 1 and 269 in phase 3, overall clinical characteristics being comparable in the two groups (**Table 2**). Considering the total study population, 421 patients (71.7%) were female, while mean age (± SD) was 74.1 ± 14.4 years. In over 87% of cases, these were emergency hospitalizations referred by Emergency Departments, for reasons almost always unrelated to thyroid disease (except in three cases of hypothyroidism and two cases of suspected or known thyroid carcinoma). The mean comorbidity count, grouped by body system, was 2.19 + 1.55 per patient, most comorbidities (68.3%) being cardiovascular. Patients had a stated history of hypothyroidism, at the time of admission, in 74.4% of cases. In over 90% of cases, patients had primary hypothyroidism, with a ratio of about 7 to 1 between overt and subclinical forms. At the time of admission, the aetiology of the primary hypothyroidism was unknown in over half of the cases (**Table 3**). In 9.7% of cases, patients presented with central hypothyroidism, mostly related to NTI syndrome. Of the patients with a known history of hypothyroidism, not all were on replacement therapy (**Table 2**). Over 94% of the patients under treatment were taking tablets, with a mean dosage of 82.3 ± 40.3 µg/die. At the time of admission, 41.1% of the patients were taking medication that could potentially interfere with LT4 absorption: in almost all these cases, the drugs concerned were proton pump inhibitors. TSH values at the time of hospitalization showed great variability, with a mean of 11.6 ± 47.5 mUI/L. During phase 1, therapy was on target for only 23.8% and 18.2% of patients, according to the ATA and AME guidelines respectively. **Figure 1** shows distribution of TSH by age group in hypothyroid subjects treated with LT4, in phase 1 (ATA target - **Figure 1A**; AME target - **Figure 1C**) and phase 3 of the study (ATA target - **Figure 1B**; AME target - **Figure 1D**): the percentage of on-target subjects, for both ATA and AME guidelines, clearly shows a further reduction in elderly subjects. **Figure 2** shows cases of over- and undertreatment in elderly and non-elderly subjects, in phases 1 and 3 of the study (ATA target - **Figure 2A**; AME target - **Figure 2B**): elderly subjects tend to show overtreatment, rather than undertreatment.

**Table 2:** Main clinical characteristics of patients enrolled in the study, overall and subdivided by study phase (phases 1 and 3).

	Phases 1 + 3	Phase 1	Phase 3	Comparison phases 1 vs 3
**Number of patients**	587	318	269	
**Females**	421 (71.7%)	230 (72.3%)	191 (71.0%)	P = 0.885 (^)
**Age** (years; mean ± SD)	74.1 ± 14.4	74.7 ± 14.2	73.4 ± 14.7	P = 0.286 (*)
**BMI** (kg/m2) (mean ± SD)	26.1 ± 5.1	25.9 ± 5.2	26.1 ± 5.0	P= 0.711 (*)
**Referral source**				
Emergency hospitalization, from Emergency Departments	515 (87.7%)	283 (89.0%)	232 (86.2%)	P = 0.796 (^)
Scheduled hospitalization	46 (7.8%)	24 (7.5%)	22 (8.2%)	P = 0.793 (^)
Transfer from other hospital department	18 (3.1%)	5 (1.6%)	13 (4.8%)	**P = 0.027** (^)
Transfer from other hospital	5 (0.9%)	5 (1.6%)	0 (0.0%)	n.d.
Hospitalized from outpatient service	3 (0.5%)	1 (0.3%)	2 (0.7%)	n.d.
**Reasons for hospitalization**	648	353	295	
Non-specific (e.g., fever, dyspnoea)	170 (26.2%)	96 (27.1%)	74 (25.0%)	
Cardiovascular	118 (18.2%)	57 (16.1%)	61 (20.5%)	
Respiratory	74 (11.4%)	45 (12.7%)	29 (9,8%)	
Infections	62 (9.6%)	41 (11.6%)	21 (6.4%)	
GI/hepatic	57 (8.8%)	25 (7.0%)	32 (10.9%)	
Neuropsychiatric	33 (5.1%)	23 (6.5%)	10 (3.3%)	
Haematological (non-oncological)	50 (7.7%)	23 (6.5%)	27 (9,1%)	
Endocrine-metabolic (non-thyroid)	28 (4.3%)	14 (3.9%)	14 (4.7%)	
Renal	30 (4.6%)	13 (3.4%)	17 (5.7%)	
Oncological	13 (2.0%)	7 (2.0%)	6 (2,3%)	
Other	15 (2.3%)	9 (3.2%)	6 (2,3%)	
**Comorbidities**				
Number of comorbidities/patient (mean ± SD)	2.19 ± 1.55	2.22 ± 1.54	2.16 ± 1.57	(*)
Cardiovascular	401 (68.3%)	220 (69.2%)	181 (67.3%)	P = 0.831 (^)
GI/hepatic	148 (25.2%)	48 (15.1%)	100 (37.2%)	**P < 0.001** (^)
Neuropsychiatric	142 (24.2%)	82 (25.8%)	60 (22.3%)	P = 0.443 (^)
Respiratory	125 (21.3%)	66 (20.8%)	59 (21.9%)	P = 0.780 (^)
Endocrine-metabolic	125 (21.3%)	68 (21.4%)	57 (21.2%)	P = 0.963 (^)
Renal	105 (17.9%)	64 (20.1%)	41 (15.2%)	P = 0.198 (^)
Haematological	57 (9.7%)	34 (10.7%)	23 (8.6%)	P = 0.428 (^)
Solid tumours	50 (8.5%)	33 (10.4%)	17 (6.3%)	P = 0.10 (^)
Rheumatological	42 (7.2%)	20 (6.3%)	22 (8.2%)	P = 0.410 (^)
Infectious	30 (5.1%)	12 (3.8%)	18 (6.7%)	P = 0.129 (^)
Other	90 (15.3%)	56 (17.6%)	34 (12.6%)	P = 0.153 (^)
**Diagnosis of hypothyroidism**				
Number of patients with known diagnosis	437 (74.4%)	242 (76.1%)	195 (72.5%)	P = 0.702 (^)
Overt primary hypothyroidism	463 (78.9%)	261 (82.1%)	202 (75.1%)	P = 0.477 (^)
Subclinical primary hypothyroidism	67 (11.4%)	32 (10.1%)	35 (13.0%)	P = 0.319 (^)
Central hypothyroidism	57 (9.7%)	25 (7.9%)	32 (11.9%)	P = 0.136 (^)
**LT4 therapy at the time of admission (°)**				
Patients on LT4 therapy	413 (70.4%)	227 (71.4%)	186 (69.1%)	P = 0.805 (^)
Tablets	391 (66.6%)	219 (68.9%)	172 (63.9%)	P = 0.572 (^)
Oral solution	19 (3.2%)	7 (2.2%)	12 (4.5%)	P = 0.136 (^)
Softgel	3 (0.5%)	1 (0.3%)	2 (0.7%)	n.d.
**LT4 dosage at the time of admission** (µg/die) (mean ± SD)	82.3 ± 40.3	83.5 ± 47.5	77.5 ± 38.1	P = 0.104 (*)
**TSH at the time of admission** (mUI/L) (mean ± SD)	11.6 ± 47.5	8.6 ± 16.9	14.6 ± 65.1	P = 0.250 (§)
**Patients on absorption-hindering medication, at the time of admission**	241 (41.1%)	138 (43.4%)	114 (42.4%)	P = 0.875 (^)
Proton pump inhibitors	231 (39.4%)	128 (40.3%)	103 (38.3%)	P = 0.750 (^)
Ferrous sulphate	26 (4.4%)	15 (4.7%)	11 (4.1%)	P = 0.724 (^)
Calcium carbonate	22 (3.7%)	9 (2.8%)	13 (4.8%)	P = 0.221 (^)

Values expressed as the number of patients (%), unless stated otherwise.

(*) = Student’s t-test for unpaired data; (§) = Mann-Whitney test for unpaired data; (^) chi^2^ test; (°) one patient on both tablets and oral solution; bold values = statically significant difference

n.d., not determined.

SD, standard deviation; BMI, body mass index; L-T4, levothyroxine; TSH, thyroid stimulanting hormone.

**Table 3:** Aetiology of hypothyroidism.

Aetiology	Number of patients (%)
**Primary hypothyroidism (overt + subclinical)**	530 (90.3%)
Unknown	282 (48.0%)
Chronic autoimmune thyroiditis	127 (21.6%)
Thyroidectomy	100 (17.0%)
Medication	12 (2.0%) (7 amiodarone, 2 lithium, 3 other medication)
Radioactive iodine therapy	3 (0.5%)
Subacute thyroiditis	3 (0.5%)
Lymphocytic thyroiditis	2 (0.3%)
Congenital forms	1 (0.2%)
**Central hypothyroidism**	57 (9.7%)
From hypothalamic-pituitary disease	5 (0.9%)
NTI syndrome	52 (8.9%)

### Results for Selected Indicators

Statistical analysis covered 22 indicators: 7 at the time of admission; and 15 post-admission, with the patient already under the IM specialist’s management. Detailed results are shown in **Table 4**.

**Table 4:** Indicators in phase 1 and phase 3, in the training group (TG) and control group (CG).

	Recommendation	Timing of assessment	Satisfactory(TG phase 1)	TGphase 1	TGphase 3	CGphase 1	CGphase 3	TG phase 3 vs TG phase 1,p value	TG phase 3 vsCG phase 3,p value	At time of admission vs post-admission
1.1	Use of LT4 as replacement therapy	On admission	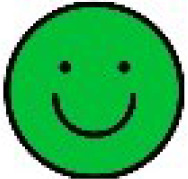	118/118 (100%)	106/106 (100%)	108/108 (100%)	81/81 (100%)	1 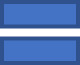	1 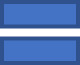	
1.2.1	Non-use of LT4 sg or o.s. in patients on absorption-hindering medication or who do not respect timing in relation to meals (ATA 2014)	On admission	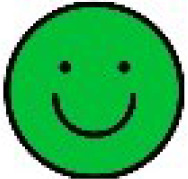	82/84 (97.6%)	82/89 (92.1%)	81/85 (95.3%)	68/76 (89.5%)	0.169 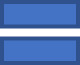	0.595 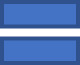	
1.3.1	Patients on TSH replacement therapy in line with ATA target	On admission	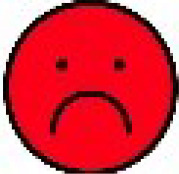	21/99 (21.2%)	21/85 (24.7%)	21/77 (27.3%)	19/69 (27.5%)	0.317 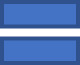	0.159 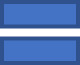	
1.3.2	Patients on TSH replacement therapy in line with AME target	On admission	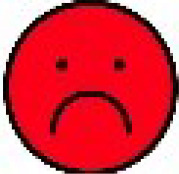	16/99 (16.2%)	12/85 (14.1%)	16/77 (20.8%)	11/71 (15.5%)	0.148 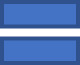	0.058 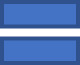	
1.4	Measurement of body weight to define LT4 dosage	On admission	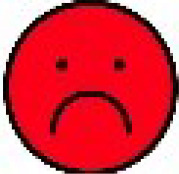	53/128 (41.4%)	52/110 (47.3%)	52/115 (45.2%)	51/86 (59.3%)	0.853 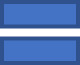	0.094 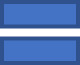	
1.5.1	Taking LT4 tbts at least 60’ before breakfast or 3h after dinner	On admission	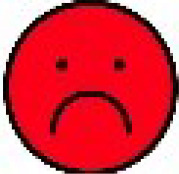	46/116 (39.6%)	25/101 (24.7%)	16/103 (15.5%)	62/70 (88.6%)	0.196 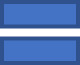	< 0.001 (*) 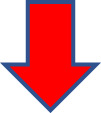	
1.5.2	Dose of absorption-hindering medication at least 4h after LT4 tbts	On admission	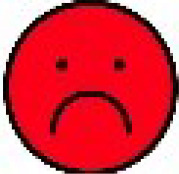	19/61 (31.1%)	4/51 (7.8%)	11/55 (20.0%)	7/45 (15.5%)	0.002 (*) 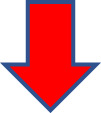	0.338 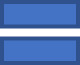	
2.1	Use of LT4 replacement therapy	Post-admission	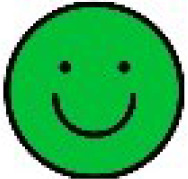	144/144 (100%)	113/113 (100%)	118/118 (100%)	105/105 (100%)	1 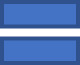	1 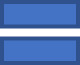	1 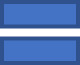
2.2.1	Non-use of LT4 sg or o.s. in patients on absorption-hindering medication or not adhering to timing in relation to meals (ATA 2014)	Post-admission	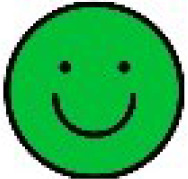	83/89 (93.2%)	54/75 (72.0%)	80/104 (76.9%)	64/75 (85.3%)	< 0.001 (*) 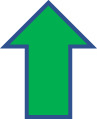	< 0.001 (*) 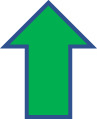	< 0.001 (*) 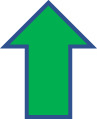
2.2.2	Use of LT4 sg or o.s. in patients not adhering to timing in relation to meals, or with absorption-hindering medication/disease (AME 2016)	Post-admission	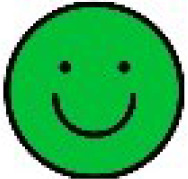	6/91 (6.6%)	17/68 (25.0%)	25/102 (24.5%)	13/76 (17.1%)	0.015 (*) 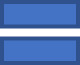	0.244 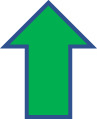	
2.2.3	Management of switches between LT4 brands/generics/formulations	Post-admission	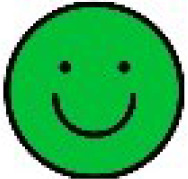	100/117 (85.5%)	62/86 (72.1%)	34/103 (33.0%)	57/66 (77.3%)	< 0.001 (*) 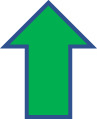	0.034 (*) 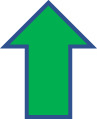	
2.5.1	Taking LT4 tbts at least 60’ before breakfast or 3h after dinner	Post-admission	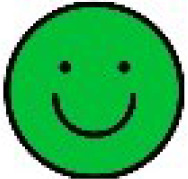	81/138 (58.7%)	50/92 (54.3%)	26/102 (25.5%)	33/93 (35.5%)	0.425 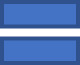	< 0.001 (*) 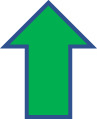	< 0.001 (*) 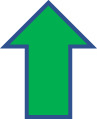
2.5.2	Dose of absorption-hindering medication at least 4h after LT4 tbts	Post-admission	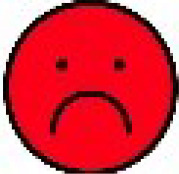	13/71 (18.3%)	9/46 (19.6%)	10/62 (16.1%)	15/49 (30.6%)	0.865 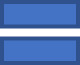	0.216 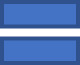	0.099 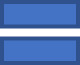
2.5.3	Investigation for absorption-hindering disease in patients requiring high doses of LT4	Post-admission	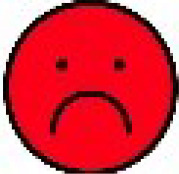	3/17 (17.7%)	0/4 (0%)	0/8 (0%)	1/4 (25%)	1 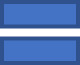	1 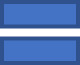	
2.6	Initial LT4 therapy < 50 mcg/die in elderly and/or cardiopathic patients and those with newly diagnosed hypothyroidism	Post-admission	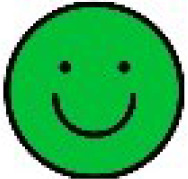	10/13 (76.9%)	3/7 (42.8%)	9/9 (100%)	5/9 (55.5%)	0.173 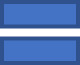	1 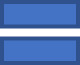	
2.7.1	Prescription of TSH after 4-6 weeks in newly diagnosed patients starting LT4	Post-admission	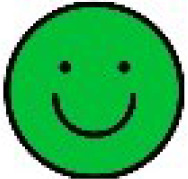	19/26 (73.1%)	3/10 (30.0%)	5/21 (23.8%)	5/24 (20.8%)	0.026 (*) 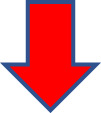	0.667 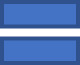	
2.7.2	Prescription of TSH after 4-6 weeks in patients changing LT4 dosage	Post-admission	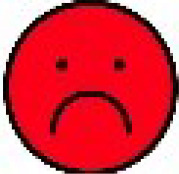	19/51 (37.2%)	11/35 (31.4%)	9/57 (15.8%)	0/24 (0%)	0.310 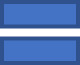	0.002 (*) 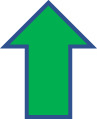	
2.7.3	Prescription of TSH after 4-6 weeks in patients switching LT4 brand/generic/formulation	Post-admission	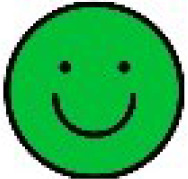	4/6 (66.7%)	22/38 (57.9%)	4/12 (33.3%)	2/7 (28.6%)	1 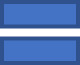	0.225 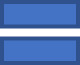	
2.8.1	Recent or post-admission TSH assay for inpatients with known history of hypothyroidism	Post-admission	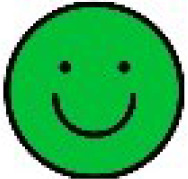	83/129 (64.3%)	85/107 (79.4%)	79/112 (70.5%)	75/85 (88.2%)	0.011 (*) 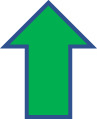	0.104 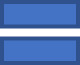	
2.8.2	Do not start LT4 or LT3 therapy for inpatients with NTI syndrome	Post-admission	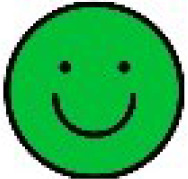	21/22 (95.4%)	23/38 (60.5%)	12/14 (85.7%)	11/12 (91.7%)	0.003 (*) 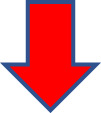	0.074 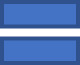	
2.9.1	No immediate prescription of LT4 in newly diagnosed subclinical hypothyroidism	Post-admission	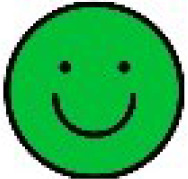	7/10 (70%)	4/10 (40%)	16/22 (72.7%)	6/21 (28.6%)	0.370 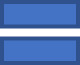	0.685 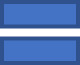	
2.9.2	Immediate prescription of LT4 i.a.w. guidelines for newly diagnosed subclinical hypothyroidism	Post-admission	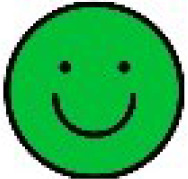	2/3 (66.7%)	6/6 (100%)	2/7 (28.6%)	9/15 (60%)	0.334 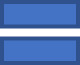	0.123 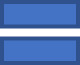	

Green = satisfactory status (at least 50% of TG patients comply with the recommendation in phase 1), or statistically significant improvement; red = unsatisfactory status, or statistically significant worsening; blue = statistically stable trend. (*) Statistically significant difference.

Regarding patients enrolled in phase 1 at IM departments assigned to the TG, an arbitrary threshold value of 50% compliance with rercommendations was present for 2/7 indicators at the time of admission (28.6%), and 12/15 post-admission indicators (73.3%).

In the TG, 2 of the 7 indicators measured basally showed significant worsening during the study: one in the comparison between phases 3 and 1 (recommended interval of at least 4 hours between taking LT4 tablets and medication interfering with their absorption), and the other in the phase 3 TG vs CG comparison (interval between LT4 tablets and meals: at least 60 minutes before breakfast, at least 3 hours after dinner).

Again in the TG, 6 of the 15 inpatient regime indicators showed significant improvement during this study. Two of these improved significantly in the phase 3 vs phase 1 comparison (non-tablet LT4 formulations for patients also receiving medication interfering with absorption, or not complying with AME recommendations on timing of replacement therapy in relation to meals; availability of recent or post-admission TSH assays in subjects with a known history of hypothyroidism); two showed an improvement in phase 3 for the TG vs CG comparison (correct timing of LT4 administration in relation to meals; prescription of TSH within 4-6 weeks, in patients with an LT4 dosage change); and the remaining two showed improvement in both the longitudinal and inter-group comparisons (reduced use of tablet formulation, so as to accommodate concomitant use of absorption-inhibiting medication or non-compliance with recommended timing in relation to meals; appropriate management of treatment change between LT4 products/formulations). The analysis also showed worsening for two inpatient regime recommendations, both in the phase 3 vs phase 1 comparison (prescription of TSH within 4-6 weeks in newly diagnosed patients starting LT4 therapy; avoidance of LT4/liothyronine (LT3) in patients hospitalized with NTI syndrome).

For four items, levels of compliance with recommendations were compared at the time of admission and subsequently, thus focusing on the impact of the IM specialist’s management. In two cases, the level of compliance with the recommendation did not change, remaining either very high (use of LT4 as replacement therapy) or low (adequate interval between LT4 dose and absorption-inhibiting medication). In the other two cases (reduced use of tablet formulation for patients on absorption-inhibiting medication, or not complying with recommended timing in relation to meals; increased prescription of the tablet formulation in patients maintaining correct timing of treatment in relation to meals), compliance increased significantly following hospitalization.

## Discussion

The FADOI-TIAMO study afforded up-to-date insights into patient characteristics and treatment modalities for hypothyroidism managed under an inpatient regime at IM departments in Italy, showing that compliance with some recommendations stated in EBGs can be improved by an EP based on outreach visits.

The general characteristics of the hypothyroid inpatients examined in this study were broadly comparable to those identified in other studies for the general population of IM inpatients in Italy, in terms of mean age, comorbidities and the clear majority of emergency hospitalizations (27). The higher number of women than men is not surprising, reflecting as it does the gender distribution of thyroid disease in the general population. About three quarters of cases presented with a known history of hypothyroidism at the time of hospitalization, the most frequent aetiological factor being chronic autoimmune thyroiditis: in about half the cases, however, the hypothyroidism was of unknown origin. This could be a reflection of the relatively low attention that IM specialists pay to this disease, even though it can be of major clinical significance when associated with comorbidities (above all cardiovascular, but also metabolical, renal, psychiatric, systemic, organ-specific or other autoimmune diseases).

In about a quarter of the patients, hypothyroidism was diagnosed at the time of hospitalization: less than half of these showed subclinical hypothyroidism.

Based on the main hypothyroidism EBGs, the study’s Scientific Board selected a set of recommendations with a view to assessing the appropriateness of disease management, both at home and for IM inpatients. Evaluation at the time of hospital admission (and thus reflecting the results of at-home treatment) identified unsatisfactory compliance with EBGs – particularly in terms of TSH targets, and timing of LT4 tablets in relation to meals and all concomitant absorption-inhibiting medication (mostly proton pump inhibitors, in some cases calcium carbonate or ferrous sulphate). A very low percentage of patients (about 20%) achieved target TSH values: this was particularly true of elderly patients, with a correspondingly higher TSH target. It should also be pointed out that target values in elderly patients are very restrictive (ATA target = 4.0-6.0 mUI/L; AME target = 4.5-5.5 mUI/L) and could be applied more flexibly in day-to-day clinical practice, bearing in mind that the relationship between TSH, age and likelihood of adverse events from overdosage (e.g., atrial fibrillation, heart failure, ischaemic heart disease, osteoporosis, fractures) is in all likelihood better expressed by continuous variables rather than categorical variables (elderly vs non-elderly, at risk vs not at risk).

Given the potential clinical impact of these departures from recommended standards, and bearing in mind that these can persist for long periods of time, it is important that IM specialists should be to the fore in addressing this issue, *inter alia* by promoting awareness among the GPs who play such an important role in home care.

Pre-training measurement of indicators showed a high degree of compliance with recommendations on LT4 monotherapy. Use of the tablet formulation was preponderant (over 80% of cases), in conditions in which this is recommended by the ATA guidelines (17) but not the AME guidelines (23). On the other hand, oral solution (o.s.) and softgel (sg) formulations were little used (16% of patients), in cases where they are recommended by the AME but not the ATA.

Measurement of the phase 1 indicators shows that the IM specialist paid little attention to a number of points regarding the management of the hypothyroid patient:

1. Body weight was measured in less than half the patients, though this – together with the aetiology of hypothyroidism – is an indispensable factor for determination of LT4 dosage. In IM departments, this becomes an even more critical factor, given the vital importance of body weight in determining dosage of other very commonly used medications (e.g., low molecular weight heparin, corticosteroids, some antibiotics);
2. Compliance with recommendations on correct timing of tablets in relation to meals and absorption-hindering medication was generally low. There was even lower compliance with recommendations on the importance of ascertaining any absorption-hindering gastrointestinal conditions in patients with high LT4 needs;
3. Similarly, there was insufficient compliance with recommendations on prescribed TSH dosage after diagnosis of hypothyroidism, and changes of LT4 dosage, brand, generic product or formulation.

However, even in the study’s pre-training phase, the IM specialists’ prescriptions were more oriented towards o.s. or sg in cases where tablets were not considered the first-choice treatment, and timing of tablets was more correct in relation to meals. This shows that the treatment prescribed by the IM specialist is both more appropriate than is the case with patients treated at home, and consistent with the most recent scientific literature (28–31).

One of the study’s main aims was to evaluate the efficacy, with a view to improved patient management, of an outreach visit-based EP. The rationale for choosing this form of training was that it is recognized as one of the most effective in influencing clinical practice (32) and has been successfully used by FADOI in other studies (18–20). One departure from these previous experiences was that the present study set a precedent in comparing compliance with standards between a group of IM centres following an EP and a CG. Results for the TG centres differed both from those obtained pre-training and from those for the CG. While the trend for 2 indicators in the TG was not good in either respect, there was significant enhancement of compliance with 6 other recommendations, particularly with regard to appropriate use of LT4 (choice of formulation, with a tendency to prescribe more o.s. and sg; timing of doses) and monitoring of TSH levels (except for cases in which LT4 therapy had just started). We consider these results meaningful, given that they were achieved with a one-shot event requiring limited expenditure and time. In addition, we cannot rule out that a more detailed EP (e.g., with distribution of training materials/information packages and/or a distance learning back-up) might have generated even clearer outcomes.

Our study presents a number of intrinsic limitations, related to its design. First, we cannot rule out that doctors’ awareness of participating in this study might have made them more attentive to the management of hypothyroidism, thus affecting results achieved in terms of compliance with recommendations (awareness bias). This concern would apply particularly to the results obtained during the post-EP phase. However, cluster randomization of centres, subdividing them between those that received an EP and those that received none, afforded a reasonably rigorous and reliable assessment of how training affected patient management. Second, retrospective data collection, based on information contained in hospital clinical record forms, might have led to some omissions in terms of information required by the study protocol. On the other hand, retrospective data collection can limit the effect of the awareness bias mentioned above, more closely aligning the information obtained to real-life clinical practice. A further limitation of the study is the impossibility of formulating realistic initial hypotheses, which meant that we were unable to make a formal calculation of the required sample size with a view to assessing departures from standard recommendations. We cannot rule out that, with a larger sample, we would have obtained further statistically significant results.

In conclusion, our study clearly demonstrates that assessment and treatment of hypothyroidism in IM inpatients (elderly subjects, often with comorbidities and polytherapy) show significant scope for improvement, The FADOI-TIAMO study, with its cluster-randomized, controlled design, is the first to show that at least some of the critical issues in application of treatment standards can be effectively addressed thanks to a standardized EP, thus determining more appropriate clinical management. Benefit could be gained from further projects, assessing whether more detailed, longer EPs than ours can generate further improvements in management of these patients, and to what extent these improvements can impact clinical outcomes.

## Data Availability Statement

The raw data supporting the conclusions of this article will be made available by the authors, without undue reservation.

## Ethics Statement

The studies involving human participants were reviewed and approved by Comitato Etico Palermo 1 - Azienda Ospedaliera Universitaria Policlinico Paolo Giaccone di Palermo. The patients/participants provided their written informed consent to participate in this study.

## List of the Members of the FADOI-TIAMO Study Group

D. Brancato, (Ospedale Civile di Partinico, Palermo); T.M. Attardo (Ospedale di Circolo e Fondazione Macchi, Varese); E. Monaco, G. Damonte (San Paolo Hospital, Savona); M. Romano, R. Romano, S. Selvaggio (Garibaldi Nesima Hospital, Catania); V. Ariete (Gubbio - Gualdo Tadino Hospital, Perugia); C. Usai (S.S. Annunziata Hospital, Sassari); M. Bigliocca (Maggiore della Carità Hospital, Novara); A. Fontanella, M. Poggiano (Fatebenefratelli Hospital, Naples); C. Alagona, M. Boaretto (San Martino Hospital, Belluno); V. Nuzzo (S. Gennaro Hospital, Naples); P. Del Duca (ASL Latina); C. Canale (Savigliano Hospital, Cuneo); F. Condemi (Bianchi-Melacrino-Morelli Hospital, Reggio Calabria); A. Cossu, L. Fiorin, L. Secchi (G.P. Delogu Hospital of Ghilarza, Oristano); A. Greco, A. Mangiacotti (Casa Sollievo della Sofferenza, San Giovanni Rotondo, Foggia); M. Nizzoli (Morgagni - Pierantoni Hospital, Forlì); M. Alessandri, G. Cati (S. Andrea Hospital of Massa Marittima, Grosseto); M. Martinelli (Castiglione del Lago Hosptal, Perugia); C. Marengo, E. Cantino, B. Deorsola (S. Croce di Moncalieri Hospital, Torino); C. Politi (F. Veneziale Hospital, Isernia); T. Ciarambino (PO Clinicizzato di Marcianise, Caserta); G. Galli, D. Carrara (Versilia Hospital of Camaiore, Lucca); A. Ciccarelli (AUSL Valle D’Aosta).

## Author Contributions

DB: project definition, Scientific coordinator of the study, outside tutor of educational programme, principal investigator, data analysis, paper writing. BB: data analysis, paper writing, member of Scientific Board of the study. TA: project definition, member of Scientific Board of the study, outside tutor of educational programme, principal investigator. AF: outside tutor of educational programme,member of Scientific Board of the study. MN:outside tutor of educational programme, member of Scientific Board of the study and principal investigator. RV: member of Scientific Board of the study. EM: principal investigator. RR: principal investigator. VA: principal investigator. CU principal investigator. EZ: project definition, project and data management, paper writing. MC: member of Scientific Board of the study. All authors contributed to the article and approved the submitted version.

## Conflict of Interest

The authors declare that the research was conducted in the absence of any commercial or financial relationships that could be construed as a potential conflict of interest.

## Publisher’s Note

All claims expressed in this article are solely those of the authors and do not necessarily represent those of their affiliated organizations, or those of the publisher, the editors and the reviewers. Any product that may be evaluated in this article, or claim that may be made by its manufacturer, is not guaranteed or endorsed by the publisher.
